# Dataset of vibrational and acoustic measurements for squeal analysis from the laboratory brake setup Friction-Induced Vibration and noisE at Ecole Centrale de Lyon (FIVE@ECL)

**DOI:** 10.1016/j.dib.2018.09.083

**Published:** 2018-10-03

**Authors:** J.-J. Sinou, D. Lenoir, S. Besset, F. Gillot

**Affiliations:** a Laboratoire de Tribologie et Dynamique des Systèmes, UMR CNRS 5513, Ecole Centrale de Lyon, 36 avenue Guy de Collongue, 69134 Ecully Cedex, France; b Institut Universitaire de France, 75005 Paris, France

## Abstract

This data article comprises raw records to investigate the dynamic behavior of a brake system during controlled braking tests and its squeal characterization through the generation of friction-induced vibration. Experiments have been performed on the laboratory brake setup Friction-Induced Vibration and noisE at Ecole Centrale de Lyon (FIVE@ECL), France.

The data provided include measurements for each component of the brake system (i.e. the acceleration measurements on the two pads and the caliper and the normal displacements of the disc), as well as a complete measurement of sounds and squeal noise in near-field and in far-field during a braking test. Data of the four operational parameters (i.e. the application pressure during braking, the rotating speed of the disc, the motor torque and the temperature close to the pad/disc brake system) are also captured during experiment.

All the results from this data will help researchers and engineers in proper analysis of brake squeal and advanced understanding of links between friction-induced vibration and squeal noise. One of the main original contribution is also to share the data sets to give the opportunity to researchers for testing and validating numerical models of brake system with the proposed data of squeal noise.

This Data in Brief article is an additional item directly along side the following paper published in the Elsevier journal Mechanical System and Signal Processing: J-J. Sinou, D.Lenoir, S. Besset and F. Gillot, Squeal analysis based on the laboratory experimental bench “Friction-Induced Vibration and noisE at Ecole Centrale de Lyon (FIVE@ECL)", Mechanical Systems and Signal Processing, Volume 119, 2019, Pages 561-588. 10.1016/j.ymssp.2018.07.006.

**Specifications table**

**Table:** 

Subject area	*Mechanical engineering*
More specific subject area	*Vibration and acoustics, friction-induced noise and vibration, brake squeal noise*
Type of data	*.mat files and ASCII files
How data was acquired	*All the dynamic signal generation and acquisition are performed via the data acquisition platform Compact DAQ from National Instrument that control the timing, synchronization, and data transfer between the Sensor-Based Input/Output modules and the external host*
Data format	*Raw*
Experimental factors	*Investigation of friction-induced vibration and squeal noise during a braking test*
Experimental features	*The data give acceleration of the two pads and caliper, displacement on the disc and squeal sounds versus time. The sampling rate of each channel of the data acquisition system is 25,600* *S s*^*-1*^
Data source location	*Data obtained from the laboratory brake setup Friction-Induced Vibration and noisE at Ecole Centrale de Lyon (FIVE@ECL), Ecole centrale de Lyon, Ecully, France*
Data accessibility	*The data are available in this article and as a supplementary file and can be found in the online version at*https://doi.org/10.1016/j.dib.2018.09.083.
Related research article	*This data is supplementary to article: J-J. Sinou, D.Lenoir, S. Besset and F. Gillot, Squeal analysis based on the laboratory experimental bench “Friction-Induced Vibration and noisE at Ecole Centrale de Lyon (FIVE@ECL)”, *Mechanical Systems and Signal Processing*, Volume 119, 2019, Pages 561-588*. 10.1016/j.ymssp.2018.07.006.

**Value of the data**

● The database provides insights of vibrational measurements for each component of the brake system such as the two pads, the disc and the caliper, as well as the radiated acoustic on near and far-fields.
● The data could be useful for researchers and industrials in understanding of brake squeal.
● The dataset can be used to undertake links between friction-induced vibrations and squeal noise.
● The database gives the opportunity to researchers for comparing and validating numerical models of brake system with the proposed data.

## 1 Data

This dataset is provided as supplementary material in a Matlab format *.mat and ASCII format. This supplementary data can be found in the online version at https://doi.org/10.1016/j.dib.2018.09.083.

The data are divided into two parts as follows:

– “time.mat” (“time.txt”, respectively) gives the value of the instant acquisition time between 0 s and 20 s in the matlab format (ASCII format, respectively),
– “data.mat” (“data.txt”, respectively) gives the vibrational and acoustic measurements as well as the evolutions of operational parameters during the braking test in the matlab format (ASCII format, respectively). Each column describes a channel measurement component produced for a specific acquisition time. Descriptions of the columns headings are provided in Table 1. The position of each channel for vibrational and acoustic measurements is provided in [1].

**Table 1:** List of the channel measurements.

ID	Name	Units	Part name
1	TriAxe.1.X	g	Rear pad
2	TriAxe.1.Y	g	Rear pad
3	TriAxe.1.Z	g	Rear pad
4	TriAxe.2.X	g	Rear pad
5	TriAxe.2.Y	g	Rear pad
6	TriAxe.2.Z	g	Rear pad
7	TriAxe.3.X	g	Front pad
8	TriAxe.3.Y	g	Front pad
9	TriAxe.3.Z	g	Front pad
10	TriAxe.4.X	g	Front pad
11	TriAxe.4.Y	g	Front pad
12	TriAxe.4.Z	g	Front pad
13	TriAxe.5.X	g	Caliper
14	TriAxe.5.Y	g	Caliper
15	TriAxe.5.Z	g	Caliper
16	Micro.1	Pa	Antenna (FF)
17	Micro.2	Pa	Antenna (FF)
18	Micro.3	Pa	Antenna (FF)
19	Micro.4	Pa	Antenna (FF)
20	Micro.5	Pa	Antenna (FF)
21	Micro.6	Pa	Antenna (FF)
22	Micro.7	Pa	Antenna (FF)
23	Micro.8	Pa	Antenna (FF)
24	Micro.9	Pa	Antenna (FF)
25	Micro.10	Pa	Caliper support (NF)
26	Micro.11	Pa	Antenna (FF)
27	Micro.12	Pa	Antenna (FF)
28	Micro.13	Pa	Antenna (FF)
29	Micro.14	Pa	Antenna (FF)
30	Micro.15	Pa	Caliper support (NF)
31	Proxy.1	mm	Disc
32	Proxy.2	mm	Disc
33	Proxy.3	mm	Disc
34	Proxy.4	mm	Disc
35	Rotation speed	rpm	–
36	Torque	Nm	–
37	Brake pressure	bar	–
38	Brake temperature	°C	–

The general description and characteristics of the laboratory brake system as well as the experimental set-up methodology are fully described on the article [1].

The following paragraph briefly describes the vibrational and acoustic measurements in link with the provided dataset.

Considering more specifically the vibrational measurements, the complete list of the experimental device is as follows:

– Four miniature triaxial Integrated Electronic Piezo-electric (IEPE) accelerometers on the two pads (ref. Bruël & Kjaer Miniature Triaxial DeltaTron@Accelerometer - Type 4520);
– One miniature triaxial Integrated Electronic Piezo-electric (IEPE) accelerometers placed on the brake caliper (ref. Bruël & Kjaer Miniature Triaxial DeltaTron@Accelerometer - Type 4520);
– Four proximitor sensors to perform non-contact measurement on the rotational disc (ref. Bently Nevada 3300 XL NSv).

Considering more specifically the acoustic measurements, the complete list of the experimental device is as follows:

– One antenna, composed of thirteen microphones, and placed in front of the brake system to perform measurements in far-field in the direction normal to the brake system (ref. Bruël & Kjaer 20 kHz Prevision Array Microphone - Type 4958);
– two microphones placed close to the brake system to perform measurements in near-field (ref. Bruël & Kjaer 20 kHz Prevision Array Microphone - Type 4958).

Finally, four operational parameters can be captured during experiment: the application pressure during braking, the rotating speed of the disc, the motor torque and the temperature close to the pad/disc brake system.

## 2 Experimental design, materials, and methods

The methodology to produce the data here presented is fully described on the article [1]. The following paragraph briefly describes the experimental protocol. The controlled braking test process is performed a time interval of 20 s as follows:

– Phase 1 for *t* = [0;3.1]s: the brake system runs at a constant rotational speed of 203 rpm without braking. This first phase allows identifying the initial test control level without braking.
– Phase 2 for *t* = [3.1;6.7]s: a rise in brake pressure is achieved up to a final constant value of 9.2 bars. This second phase corresponds to a transient braking behavior before the controlled braking test.
– Phase 3 for *t* = [6.7;20]s: the controlled braking test with the stabilization of the rotational speed at 203 rpm is operational. Squeal characteristics based on vibrational and acoustics measurements can be performed on this last phase.

The time evolutions of the four operational parameters (i.e. the speed rotation of the disc, the motor torque, the brake pressure and the temperature) during the three phases are available in Fig. 6 of [1].

This data article comprises raw records for *t* = [0;20]s in order to investigate the dynamic behavior of a brake system during the three phases of the controlled braking test and its squeal characterization through the generation of friction-induced vibration.

All results from this raw records are analyzed and discussed in [1] for brake squeal characterization through the generation of friction-induced vibration and noise.
